# Drug repositioning for idiopathic epilepsy using gene expression signature data

**DOI:** 10.6026/97320630018845

**Published:** 2022-10-31

**Authors:** Pawan Kumar, Deepak Sheokand, Annu Grewal, Vandana Saini, Ajit Kumar

**Affiliations:** 1 Toxicology and Computational Biology Group, Centre for Bioinformatics, Maharshi Dayanand University, Rohtak, Haryana, 124001

**Keywords:** Gene-expression study, Gene expression omnibus, Connectivity map, Voltage-gated calcium channel, GABA receptor, Molecular docking study, homology modelling

## Abstract

Epilepsy is one of the most common neurological disorders, affecting millions of patients with a substantial economic and human burden. About 30-40% of epileptic patients remain un-treated after the therapeutic option. Genetic or idiopathic epilepsy count about 40% of total epilepsy patients, showing a maximum percentage for drug-resistant epilepsy. Since the last century basic approach to understanding disease progression and drug discovery has been through the prism, exploring all possible causes and treatment options. Here we report about the gene expression-based drug repositioning study for epilepsy. Epilepsy gene expression data was retrieved from the Gene Expression Omnibus database, while drugs-associated gene expression data was retrieved from the Connectivity map (CMAP). The study predicted309 drug compounds which can alter genetic epilepsy-mediated gene signature using an in-house developed R-script. These compounds were docked against identified epilepsy targets– Voltage-gated sodium channel subunit α2 (Nav1.2); GABA receptor α1-β1; and Voltage-gated calcium channel α1G (Cav3.1)using Carbamazepine, Clonazepam, and Pregabalin as standard drugs, respectively. Twenty-one predicted drug compounds showed better binding affinity than respective standards against the selected epileptic receptors. Among these drug compounds, Ergocalciferol, Oxaprozin, Flunarizine, Triprolidine and Cyproheptadine have been previously reported for anti-epileptic activities and can be potential hits to target idiopathic epilepsy.

## Background:

Epilepsy is one of the lifetime-associated neurological disorders with a high prevalence worldwide [1,2]. The severity of the disease can be estimated with the factor that about 1/3rd of patients remain unresponsive to the available therapeutic options. The main reason behind this may be the multi factorial nature of the disease and insufficient information about the exact cause of epileptic seizures in patients [3]. Hence, most patient’s suffer chronic side effects without a satisfactory response. Various metabolic and structural disorders are primarily associated as the main epilepsy-causing factors occur due to genetic alterations or physical injury to the brain. Advancements in genetic testing revealed the genetic basics of epilepsy in more than half of neonatal and childhood epilepsy cases [4]. Genetic-caused epilepsy, also known as the idiopathic epilepsy type, accounts for >40% of all epilepsy types [5]. Accounting research has focused on nucleotide polymorphism and the related response of associated ion channels and metabolic regulators. As early as 400BC, Hippocrates suggest the genetic nature of epilepsy [6]. But in modern science, the first study of the hereditary nature of epilepsy proceeded in 1995 with the identification of missense mutation of neuronal nicotinic acetylcholine receptor α4 subunit (CHRNA4) in familial nocturnal frontal lobe epilepsy [7]. Advancement in gene sequencing and genome-wide association studies (GWAS) reveals voltage-gated sodium channels (VGSCs), neuronal potassium channels (KCNs), calcium channels, and GABA receptors as essential lepilepsy-associated genes [8, 9]. Genetic variations in the potassium channel subfamily D3 (KCND3), glutamate NMDA type receptor subunit 1 (GRIN1), VGSC α1 (SCN1A), and hyper polarisation-activated cyclic nucleotide-gated potassium channel 1 (HCN1) were identified to be major contributing factors for the Dravet syndrome [10, 11]. Other epilepsy conditions with myoclonic-atomic seizures, Landau-Kleffner, Lennox-Gastaut and pyridoxine-dependent epilepsy types have reported mutations in various genes, including cyclin-dependent kinase-like 5 (CDKL5), potassium voltage-gated channel subfamily Q member 2 (KCNQ2), SCN1A, SCN2A, SCN3A, aristaless related homeobox, solute carrier family 2 member 1 (SLC2A1), SLC6A1, GRIN1, DNA polymerase subunit γ (POLG1), Neurexin 1 (NRXN1), GABA receptor α1 (GABRA1), GABRA6, GABA receptor γ2 (GABRAG2), GABA receptor β3 (GABRB3), Casein kinase 2α (CSNK2A1),Cytochrome P450 family 2 sub-family C member 9 (CYP2C9), CYP3A4, and Chromatin helicase DNA binding protein 2 (CHD2) [5, 10, 12, 13]. Gene expression profiles are valuable resources storing gene expression change during a diseased condition, representing responsible genes for hereditary causes of disease. The altered gene expression needs to be normalised to maintain regular gene expression resulting in a healthy phenotype. In opposite, drugs also affect gene expression profiles and can be studied to revert the altered gene expression signature of the disease condition. Thus the identified drug reversing gene expression pattern of disease conditions got a new indication and will be marketed in no time due to available safety data from previous clinical trials. Previously, various studies successfully predicted repositioned drug compounds based on their gene expression signature reversal profile for CNS disorders [14], Alzheimer's disease [15, 16], cancer [17, 18], SARS COVID-19 [19] and other disease conditions. In this study, we first identified gene expression changes in idiopathic epilepsy patients from the data obtained from gene expression omnibus (GEO).CMAP (connectivity map) drug library was screened against the epilepsy gene expression profile to check their potency to reverse the gene expression pattern. Screened drugs were further molecular docked against previously identified epilepsy targets voltage-gated sodium channel subunit α2 (Nav1.2), GABA receptor α1-β1, and voltage-gated calcium channel α1G (Cav3.1) [20]. Twenty-one compounds showed better binding affinity than standard drugs against selected receptors. These compounds showed to reverse the gene expression pattern observed in epilepsy, along with better binding affinity against epileptic receptors. Hence, these compounds can be potentially repositioned drugs while further in-vivo studies can validate their anti-epileptic properties.

## Material and Methodology:

### Collection of epilepsy-specificgene expression data:

Rawat et al. [21] have previously studied microarray-based gene expression data of 75 individuals, including 34 epileptic patients and 41 healthy controls. These epileptic patients were further classified based on epilepsy types, idiopathic, cryptogenic and symptomatic, counting 13, 09, and 12 patients, respectively. The data was grouped into 3 groups based on the epilepsy types a) idiopathic vs healthy, b) cryptogenic vs healthy, and c) symptomatic vs healthy. In their study, Idiopathic epilepsy showed a significant change in gene expression count showing 274 upregulated genes and 388 down regulated genes (Table 1 - see PDF). In comparison, idiopathic other studied epilepsy types showed ~100-150 differential regulated genes. Hence, idiopathic epilepsy types correlate with patients' gene expression change and epilepsy symptoms. The same gene expression data was retrieved from the GEO database with ID: GSE143272.The data were re-screened using the GEO2R tool to re-analyse the gene expression changes between the three groups keeping the default parameters of fold change (FC >1.3) and a high level of significance (p < 0.1).

### Retrieval of drug perturbation data and gene expression comparison:

Drug perturbation or drug-associated gene expression data was downloaded from CMAP (CMAP_2016) using the "downloadPertSig" function of the "PharmacoGx" library within the R language programming script. Using the in-house developed R script, differential epileptic gene expression signature data was compared with the drug perturbation data to predict a connectivity score between the two. The connectivity score represents the association between drug-gene and disease gene expression change and the potency to reverse the gene expression pattern. The final result was sorted based on the connectivity score. Drugs with negative and zero connectivity scores were chosen to be repositioned for epilepsy treatment based on the drug-and disease-associated gene expression-based data.

### The market status and blood-brain barrier permeability prediction of selected drugs:

Predicted gene-expression-based repositioned drugs were checked for their market approval status from the drug bank database. Approved and nutraceutical drugs were kept for future study, while experimental, investigational and withdrawn drugs were removed due to drug safety issues. Selected drugs were checked for their blood-brain barrier (BBB) permeability. BBB is a highly selective semipermeable border to regulate molecular movement between blood and the brain. BBB permeability was predicted using the in-house developed web-server tool "BBBper". Drug compounds predicted to cross BBB were selected for further docking studies.

### Selection and preparation of tertiary structures of epileptic target receptors:

A multi-target docking approach was used in the present investigation. Previously identified 3 primary epilepsy targets, namely, VGSC α2 (Nav1.2), GABA receptor α1-β1 (GABAr α1-β1), and VGCC α1G (Cav3.1), were selected for this study [20].The tertiary structure of target receptors Nav1.2, GABA receptor α1, and Cav3.1 were retrieved from the PDB database with PDB IDs: 6J8E-A, 6HUJ-A, and 6KZP, respectively. But the tertiary structure of GABA receptor β1 was unavailable in the PDB database, so it was generated by homology modelling using the Swiss model web server, taking 6HUJ-B as a template. The modelled structure was validated using the QMEAN score [22], Ramachandran Z-score, Verify 3D [23], and Ramachandran plot [24]. To form the GABA receptor α1-β1 complex for further studies, the tertiary structures of GABA receptor α1 and modelled GABA receptor β1 were docked together using the Hex8.0.0 [25]. Finally, all the structures of selected target receptors were energy minimised using UCSF Chimera v1.5 [26] to normalise the net inter-atomic force on each atom close to zero.

### Molecular docking study:

Selected drugs were virtually screened using Autodock v4.2.6 [27, 28] against selected epilepsy targets Nav1.2, GABA receptor α1-β1, and Cav3.1. PDB files of target proteins were converted to pdbqt format using the Autodock tool after the assignment of Kollman and Gasteiger charges. Molecular files of selected drug compounds were downloaded from the Drug Bank database [29] and converted to pdbqt file format. Grid box parameters (Table 2 - see PDF) for binding pockets were saved as grid parameter files (GPF) for each protein receptor, and necessary map files were generated after the autogrid run. Molecular docking was performed using the Lamarckian Genetic Algorithm as a search parameter, and 100 independent runs with a step size of 0.2Å for translation were performed. The maximum number of gestations was set to 1000, and the maximum number of top individuals that automatically survived was set to 1 with a mutation rate of 0.02, crossover rate of 0.8, cluster tolerance of 0.5Å and external grid energy of 1000. Top marketed anti-epileptic drugs (AEDs): carbamazepine, clonazepam and pregabalin were selected as standard drugs for epilepsy receptors Nav1.2, GABA receptor α1-β1, and Cav3.1, respectively [20].

## Results:

### Collection of epilepsy-specific gene expression data:

Gene expression signature data of 41 healthy individuals and 34 epileptic patients (Idiopathic: 13; cryptogenic: 09; and symptomatic: 12 - see PDF) was retrieved from the GEO database.The data was screened for expression changes in 4 groups: all epilepsy patients vs healthy, idiopathic vs healthy, cryptogenic vs healthy and symptomatic vs healthy and studied using the GEO2R tool available at NCBI-GEO. As to the previous result of Rawat et al. [21] idiopathic epilepsy showed a significant change in gene expression count leading to most differential regulated genes (Figure 1 - see PDF). So, gene expression data of idiopathic epilepsy types vs healthy was selected for further screening (Suppl file: 1 - see PDF).

### Retrieval of drug perturbation data and gene expression comparison:

Drug perturbation data was available for marketed drugs in a single package (CMAP_2016) hosted by CMAP and was downloaded using the "downloadPertSig" function of the "PharmacoGx" library in the R language. Previously analysed differential idiopathic epilepsy vs healthy gene expression signature data was compared with the drug perturbation data using an in-house developed R-script (Suppl file: 2 - see PDF) to predict a connectivity score between drug and epilepsy-associated genes. Connectivity map data was screened for already marketed AEDs available at the CMAP_2016 library. Nine marketed AEDs showed connectivity scores concerning epileptic gene expression change (Table 3 - see PDF). AEDs trimethadione, topiramate and carbamazepine showed negative connectivity scores confirming their role in targeting idiopathic and absence epilepsy type [30-33], while widely marketed AEDs acetazolamide, primidone, ethosuximide, valproate and vigabatrin showed neutral (zero) connectivity score, correlating no therapeutic for genetic epilepsy types, but in-vivo and clinical studies of these drugs showed a therapeutic response in genetic epilepsy type [34, 35]. AED gabapentin showed a positive connectivity score, representing the role of gabapentin with epilepsy-associated gene expression patterns. Additionally, literature data mining also states no therapeutic option for gabapentin in idiopathic, absence or drug-resistant epilepsy [36]. Hence, drugs showing negative and zero connectivity scores were picked for further study. Overall, 1219 drug compounds showed a connectivity score, including 309 drugs showing a negative connectivity score means these drugs can alter the gene expression of epileptic conditions. The maximum number of 690 drug compounds showed no correlation with a connectivity score of 0, and 220 drugs showed a positive connectivity score, stating their role in promoting epilepsy-like gene expression change. Drugs with negative connectivity appear to reverse gene expression patterns, while drugs showing zero connectivity score might act as non-genetic epilepsy therapeutic like marketed AED valproate. Hence,999 drugs showing either a negative or zero connectivity score were selected as potential repurposed drugs targeting non-genetic and genetic types of epilepsy (Supplementary file: 3a - see PDF).

### The market status and blood-brain barrier permeability prediction of selected drugs: 

Predicted 999gene-expression-based repositioned drugs were checked for their market approval status from the Drug Bank database. Only 612 drugs have reported status, among which 423 drugs were labelled approved, 11 were nutraceutical, 114 drugs were experimental and investigational, 55 were withdrawn, and for 9 drugs, no status data was available on Drug Bank (Supplementary file: 3b - see PDF). With available safety data, approved and nutraceutical drugs were kept for future study, counting 434 drugs, which were further checked for their BBB permeability prediction using BBBper. The BBBper predicted 323drug compounds (102 with negative connectivity and 221 with zero connectivity scores) to cross BBB and were selected for further docking studies (Supplementary file: 3c - see PDF).

### Epilepsy targets proteins and their tertiary structure:

From our previous study, we have concluded VGSC α2 (Nav1.2), GABA receptor α1-β1 (GABAr α1-β1), and VGCC α1G (CAV3.1) as major epilepsy target proteins for multi-targeted epilepsy therapy [20]. Protein tertiary structures of Nav1.2, GABA receptor α1, and Cav3.1 were available on the PDB database with PDB IDs: 6J8E-A, 6HUJ-A, and 6KZP, respectively. The tertiary structure of GABA receptor β1 was unavailable on the PDB database and was homology modelled from the Swiss model web-server, taking 6HUJ-B as a template (Figure 2a - see PDF).The template shows 54 % sequence similarity and coverage of 78.28 %.The modelled structure showed a QMEAN score of -2.88, Ramachandran Z-score of -2.818, and a pass verified 3D status. Ramachandran plot (Figure 2b - see PDF) analysis showed 93.4 % reside under favoured reason and the remaining 6.6 % residue in the allowed area representing a good predicted model of GABA receptor β1 (Table 4 - see PDF).The tertiary monomeric structures of GABA receptor α1 and GABA receptor β1 were docked using Hex tool to form a GABA receptor α1-β1 complex. This docked complex showed binding energy of -1206.05 KJ/mol and was selected for further docking study after energy minimisation (Figure 2c - see PDF). All the selected epilepsy receptor files were energy minimised using UCSF Chimera v1.5.

### Molecular docking study:

The molecular docking study was done using Autodock v4.2.6.Standard drugs carbamazepine, clonazepam, and pregabalin showed binding energy of -7.13, -6.14 and -5.76 Kcal/Mol against epileptic receptors Nav1.2, GABA receptor α1-β1, and Cav3.1, respectively.128, 59, and 242drug compounds showed better binding energy than standard drugs against epilepsy receptor Nav1.2, GABA receptor α1-β1, and Cav3.1, respectively (Supplementary file: 3d).Overall screening against all three receptors resulted in 41 drugs (21 with negative connectivity and 20 with zero connectivity scores) having better binding energy than standards (Table 5 - see PDF).

## Discussion:

Epileptic gene expression signature change data is seldom available in the GEO database. Available microarray data was screened for differently expressed gene information using the GEO2R program at GEO with a high significance level. Drug gene expression data was retrieved from the CMAP library and screened against epilepsy gene expression signature to obtain connectivity score of drugs representing their potency to revert gene signature. Three hundred nine drugs showed a negative connectivity score, and 690 drugs represented a neutral or zero connectivity score. These 999 drugs were predicted to revert gene expression patterns. Predicted 999 drugs were checked for their market approval status and BBB permeability prediction. The market approval status of 608 drugs was available on the Drug Bank database, concluding that 423 were approved, 11 nutraceuticals, 114 were experimental and investigational, and 55 were withdrawn drugs. Experimental-investigational and withdrawn drugs lacked safety information and were not selected for further study. Hence, 434 approved and nutraceutical drugs were selected as safe drugs for our repositioning study. Upon screening of these drugs, 323 drug compounds were predicted to be BBB permeable by BBBper, including 102 drugs with negative connectivity and 221 drugs with zero connectivity scores. These 323 predicted gene-expression-based repositioned drug compounds were molecular docked against identified epilepsy receptors: Nav1.2, GABA receptor α1-β1, and Cav3.1. Marketed AEDs carbamazepine, clonazepam and pregabalin were selected as standard drugs for epilepsy receptor Nav1.2, GABA receptor α1-β1, and Cav3.1, respectively. Conclusive 41 drug compounds showed better binding affinity than standards against the three epilepsy receptors. These compounds include 21 predicted repositioned drugs with negative and 20 with zero connectivity scores. These predicted repositioned drugs have diverse reported functions. Vitamin D3 analogue ergocalciferol has a minimum connectivity score of -0.35, with an excellent binding affinity with selected epilepsy receptors. Researchers have shown the role of Vitamin D3 supplements in reducing epileptic seizures, with a median of 40% [37]. Vitamin D3 is also involved in brain development, including cell growth, differentiation, and neuro protection. Hence, ergocalciferol can be a possible repositioned drug candidate for epilepsy treatment.

Non steroidal anti-inflammatory drug (NSAIDs) oxaprozinis also reported to have an anti-epileptic effect in rat models [38, 39]. But the other predicted NSAIDs, ketoprofen, do not show any anti-epileptic response during in-vivo studies [40]. Conclusive NSAID oxaprozin can be a repositioned drug compound as epilepsy therapeutics. Predicted drugs metixene and trihexyphenidyl are acetylcholine receptor inhibitors, which are reported to cause seizures in vitro studies [41]. The predicted repositioned drug flunarizine is a calcium channel blocker, also observed in our docking study. Due to its calcium blocker activity, various researchers have tried to check its anti-epileptic properties in the developed epilepsy model and got a significant response [42-44]. Antidepressants are used as combinational therapy options in epileptic patients observing depression-like symptoms. Still, selective serotonin uptake inhibitors (SSRIs) show anti-epileptic properties, while tricyclic anti depressants have been reported to cause seizures as a side effect in 2-3% of patients [45-47]. Hence, predicted drugs protriptyline and nortriptyline are tricyclic antidepressants and cannot be used as anti-epileptic therapeutic. Many histamine receptor antagonists have been validated in animal epilepsy models, with their anti-epileptic activity in clinical trials [48]. Hence, histamine receptor inhibitors triprolidine and cyproheptadine can be in-vivo validated for their anti-epileptic effects. Serotonin and dopamine receptor antagonists increase seizure risk by about 3% [49]. Consequently, dopamine receptor antagonist perphenazine, pimozide and serotonin receptor inhibitor pizotifen cannot be potential anti-epileptic compounds.

Conclusively, ergocalciferol, oxaprozin, flunarizine, triprolidine and cyproheptadine can be possible gene expression-based repositioned drugs. These compounds have negative connectivity scores representing a high probability of reversing altered gene expression patterns in epileptic patients. Besides this, these compounds also have a multi-targeting approach to target primary epilepsy targets. Hence, these compounds can serve as first-line repositioned anti-epileptic compounds showing therapeutic responses in genetic and non-genetic epileptic patients.

## Conclusions:

The present study about screening epilepsy gene expression profiles against the drug's gene expression signature helped predict drugs for repositioning new drugs for epilepsy treatment. The study predicted 21 drugs with negative connectivity scores and better binding energy than standard drugs against three identified epilepsy drug targets. Among them, marketed drugs ergocalciferol, oxaprozin, flunarizine, triprolidine and cyproheptadine have shown anti-epileptic-like properties in controlling seizures in earlier reports. Hence, these compounds can be potential hits against the treatment of idiopathic absence epilepsy types.
